# Anti-tubulin-alpha-1c antibody as a marker of value in Behçet syndrome

**DOI:** 10.1007/s10067-021-06025-7

**Published:** 2022-02-07

**Authors:** Mariam Maged Amin, Osama M. Abdel Latif

**Affiliations:** Department of Internal Medicine, Clinical Immunology and Allergy, Faculty of Medicine, Ain Shams University, Ramsis St., Abbasseya, Beside El Nour Mosque, 11566 Cairo, Egypt

**Keywords:** Anti-endothelial cell antibodies, Behcet syndrome, Tubulin-α-1c antibodies, Vasculitis

## Abstract

**Background:**

Behçet’s syndrome (BS) is a multi-systemic vasculitis characterized by recurrent oral ulcers, genital ulcers, ocular lesions, and other systemic manifestations. As there is no laboratory diagnostics of BS, the diagnosis is mainly clinical.

**Objective:**

To investigate the utility of the autoantibody against tubulin-α-1c in diagnosis of BS and its clinical significance.

**Methods:**

Sixty BS patients and sixty healthy controls were enrolled in this study. We assessed all patients by Behçet disease current activity form (BDCAF), routine laboratory investigations, and immunological markers (ANA, anti-DNA, ANCA). Anti-endothelial cell antibodies (AECA) and anti-tubulin-alpha-1c antibodies were performed for all participants.

**Results:**

Regarding duration of illness, Birmingham Vasculitis Activity Score (BVAS), and BDCAF, the mean value was 4.77 ± 4.239, 19.80 ± 10.020, and 9.52 ± 5.476, respectively. On comparing laboratory investigations, there was only significant increase in anti-tubulin-alpha-1c antibody in BS patients compared to healthy controls. Regarding AECA, there was no any significant correlation except with CRP. Anti-tubulin-alpha-1c detected significant direct correlation with the presence of posterior uveitis, panuveitis, and venous thrombosis as well as BVAS, C4, and protein/creatinine ratio. Regarding diagnostic performance of both AECA and anti-tubulin-alpha-1c, the cutoff value of AECA for diagnosis was 27.250, with sensitivity and specificity of 93.3% and 96.7%, respectively. The cutoff value of the anti-tubulin-alpha-1c for diagnosis was 22.300, with sensitivity and specificity of 100% and 96.7% respectively.

**Conclusion:**

Anti-tubulin-α-1c antibodies are of diagnostic value in BS and are indicative of activity with 100% sensitivity and 96.7% specificity.

**Table:** 

**Key Points** • *There is lack of specific laboratory, radiological, or histological diagnostics for Behcet syndrome.* • *We aimed to evaluate the significance of tubulin-α-1c autoantibody in diagnosis of Behcet syndrome.* • *There is elevation of tubulin-α-1c autoantibody with sensitivity and specificity of 100% and 96.7%, respectively.*

**Supplementary Information:**

The online version contains supplementary material available at 10.1007/s10067-021-06025-7.

## Introduction

Behçet’s syndrome (BS) is a syndrome of relapsing and remitting pattern in which most of its clinical presentations are due to blood vessel vasculitis (small, medium, or large sized) of both the arterial and venous system. Heterogeneity is a dominant feature of BS making its diagnosis and treatment challenging [1, 2]. Globally, it affects mostly the Eurasian populations along the ancient trading route which extends from eastern Asia to the Mediterranean basin. Egypt is considered a high incidence for being a Mediterranean country [3] with a prevalence of 3.6/100,000 [4].

BS is characterized by a triad of recurrent oral ulcers, genital ulcers, and ocular lesions with/without other clinical dermatological, cardiovascular, gastrointestinal, and neurological symptoms [5]. Due to the lack of specific laboratory, radiological, or histological diagnostics for BS, the diagnosis is mainly clinical. Different diagnostic criteria were proposed [6, 7], and the revised International Criteria for Behçet’s Disease (ICBD) 2010 are the most commonly used and validated on different ethnic groups [8].

The etiology of BS remains unknown. Several genetic factors and immune-mediated mechanisms might contribute. However, unlike other autoimmune diseases, BS cannot be diagnosed using any autoantigens as in other autoimmune diseases. The lack of reliable diagnostic markers delays improvement of treatment in BS [9, 10].

It is well established that endothelial cells are involved in vasculitis [11–13]. Hence, IgG autoantibodies to antigens expressed on endothelial cells have been detected in a variety of autoimmune diseases [14, 15]. One of these is a circulating immunoglobulin, called anti-endothelial cell antibody (AECA), detected in BS. However, no evidence exists if these antibodies can influence clinical presentation and/or activity of BS or not [14, 16, 17].

Though AECA can detect vascular damage of BS [18–20], its presence in other systemic vasculitis is quite high, making accurate diagnosis demanding. Hence, identification of novel markers for BS diagnosis is required [12, 13, 21]. For this, a diversity of autoantigens have been studied in BS, including vascular proteins such as alpha-enolase [19], organ or tissue original proteins such as alpha-tropomyosin, and selenium-binding protein [18, 20, 22], however with limited sensitivity and specificity [23].

Microtubules are cytoskeletal basic structural components of vascular cells that are assembled from α-/β-tubulin heterodimers [24]. The tubulin superfamily includes six distinct families, and they exist in several isotype forms. Tubulin-α-1c, also known as tubulin-alpha-6, is one of the alpha-tubulin family members [25]. The expression of tubulin-α-1c among different cell types is still elusive [24]. Alpha-tubulin has been identified as an autoantigen in patients with chronic allograft rejection [26, 27]. However, the clinical relevance of α-tubulin autoantibodies in autoimmune diseases is still unclear. Hence, we aimed to evaluate the significance of autoantibody against tubulin-α-1c, as a novel diagnostic marker in BS, and its possible link with BS activity and phenotypes.

## Methodology

### Study participants

A case–control study was conducted on sixty patients diagnosed to have Behçet syndrome (BS) according to the International Study Group for Behçet’s Disease classification criteria [28]. They were recruited from the Immunology Ward and Clinic of Ain Shams University Hospital. Another group of matched sixty healthy individuals was enrolled as control group. Patients with any other autoimmune diseases including juvenile BS, diabetes mellitus, cardiac diseases, thyroid dysfunction, and hepatic or renal dysfunction; smokers; those with history of infection (< 3 months); or patients receiving medications that act on endothelial cells (statins, angiotensin-converting enzyme inhibitors) were excluded from the study. The study was approved by the research ethical committee of Ain Shams University. A written informed consent was obtained from all participants.

### Study design

Detailed clinical history (including duration of illness; family history; presence of oral ulcers, genital ulcers, erythema nodosum or acneiform eruptions, arthralgia or arthritis, and vascular, pulmonary, gastrointestinal, neurological, and ophthalmic manifestations) and examination were applied for all recruited patients with assessment of disease activity and Birmingham Vasculitis Activity Score. We assessed all patients with the routine laboratory investigations (ESR, CRP, kidney and liver functions, complete blood picture, urine analysis with pr/cr ratio, C3, C4, eGFR) in addition to the immunological markers (ANA, anti-DNA, ANCA). AECA and anti-tubulin-alpha-1c were withdrawn for all participants. Some radiological assessment was done when needed as arterial and venous duplex. Ophthalmological assessment was done for all participants.

#### Pathergy test

It was performed by inserting a small 5-mm needle obliquely into the patient’s flexor aspect into the dermis forearm skin under complete sterile condition and without injecting saline of the forearm. The reaction is considered positive if either a papule, a pustule, or an ulcer forms at the puncture site within 48 h [29].

#### Behçet syndrome activity

We assessed BS activity by the Behçet Disease Current Activity Form (BDCAF) score [30]*.* It includes presence or absence of new headache, mouth ulcers, genital ulcers, skin lesions (pustules or erythema nodosum), joint involvement (arthralgia or arthritis), gastrointestinal symptoms (nausea/vomiting/abdominal pain or diarrhea with altered/frank blood), eye involvement (red or painful eye, blurred/ reduced vision), neurological involvement (difficulty with speech or hearing, blurring of/double vision, weakness/loss of feeling of arm or leg, and loss of balance), and major vessel involvement (chest pain, breathlessness, hemoptysis, and pain/swelling/discoloration of the face or arm or leg) over the last 4 weeks. The BDCAF score ranges from 0 to 12, and accordingly, the patients were divided into two groups: a higher disease activity group with a score equals to or more than 4 out of 12 and a lower disease activity group with a score less than 4.

#### Birmingham Vasculitis Activity Score (BVAS)

We calculated BVAS at time of diagnosis to assess vascular activity. The weighted score is based on symptoms and signs in nine separate organ systems. Score ranges from 0 to 63 [31].

#### Biochemical analysis for anti-tubulin-alpha-1c antibody

Samples were analyzed for anti-tubulin-α-1c autoantibodies by enzyme-linked immunosorbent assay. Ninety-six-well plates were coated with 10 μg/mL recombinant tubulin-α-1c protein in 0.25% phosphate-buffered saline (PBS). Samples were then diluted at 1:100 with PBS-T containing 1% BSA. All samples were incubated for 1.5 h at 37 °C. Then, 100 μL of goat anti-human IgG, diluted at 1:5000 in PBS-T, was added to each well and incubated for 30 min at 37 °C. The antibodies were then detected with o-phenylenediamine after washing with PBS-T. The reaction was stopped by adding 100 μL of 2 M sulfuric acid in each well.

#### Statistical methodology

Data was revised, coded, tabulated, and introduced to a PC using Statistical Package for Social Science (SPSS 20 for windows). Checking for quality of data and data entry was performed. Data was presented and suitable analysis was done according to the type of data. Descriptive statistics and mean ± standard deviation (± SD) were used for parametric numerical data, while frequency and percentage were used for qualitative data. For analytical statistics, we used Student’s *t* test to assess the statistical significance of the difference between two study group means. The chi-square test was used to examine the relationship between two qualitative variables. Fisher’s exact test was used to examine the relationship between two qualitative variables when the expected count is less than 5 in more than 20% of cells. The Pearson correlation was used to measure the strengths of association between two quantitative variables.

## Results

**Fig. 1:**
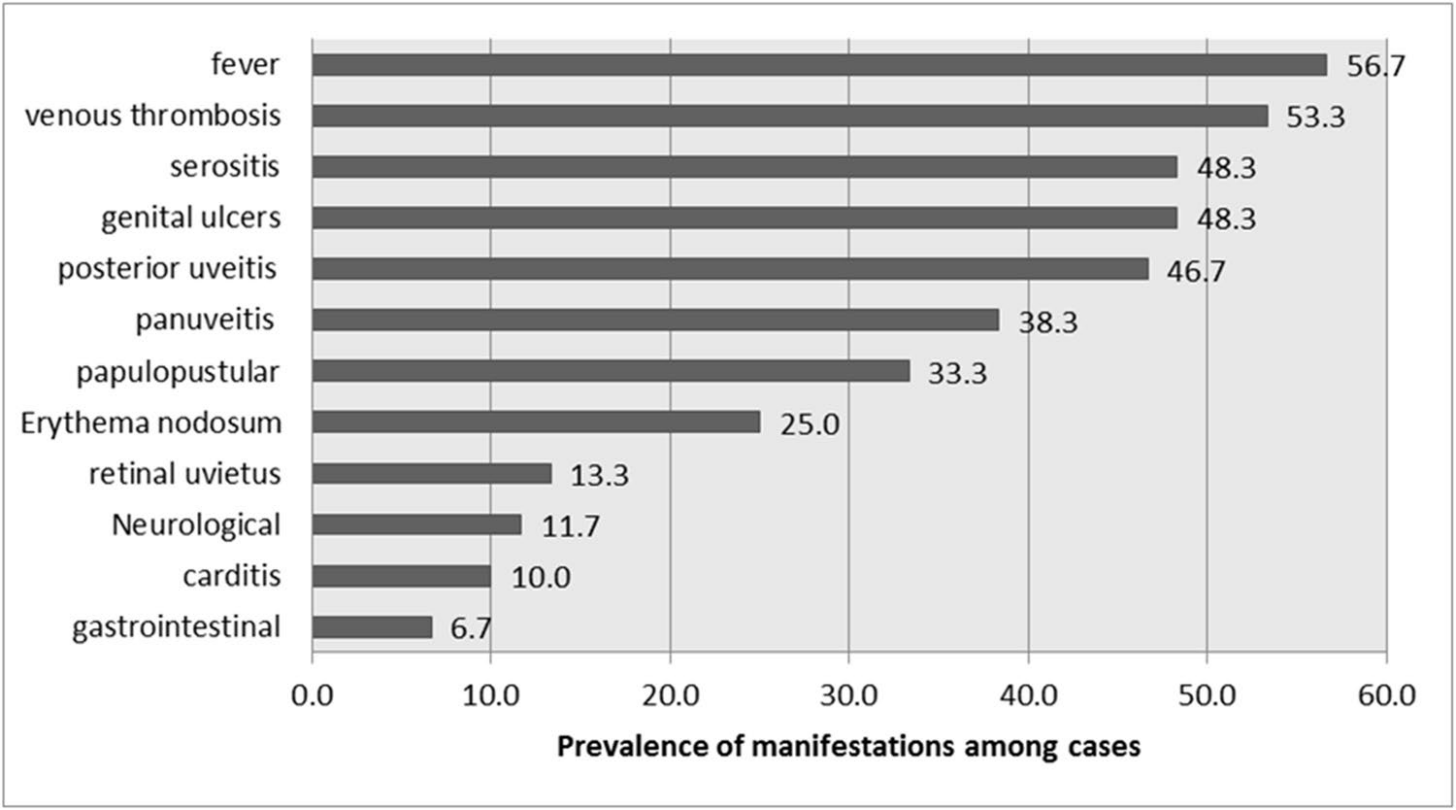
Prevalence of Behçet syndrome (BS) manifestations among patients (60 cases)

Demographic characteristics and manifestations of both studied groups are shown in Table 1. Both studied groups were comparable for age and gender. Mean age of the patients with BS and the healthy controls was 30.3 ± 5.8 and 28.9 ± 6.1 years, respectively. Mean disease duration of BS was 4.77 ± 4.239 years. Regarding BVAS score and Behcet activity score of patients, the mean value was 19.80 ± 10.020 and 9.52 ± 5.476, respectively. Figure 1 shows the prevalence of different presentations among BS patients. Fifty-five percent of BS patients had high disease activity index, and 45% had low disease activity index.

**Table 1:** Demographic characteristics and manifestations of both studied groups

		Study groups		*p* value
		Cases	Controls	
Age in years (mean ± SD)		30.3 ± 5.8	28.9 ± 6.1	0.203
Gender	Males	31 (51.7%)	31 (51.7%)	0.897
	Females	29 (48.3%)	29 (48.3%)	
Smoking	Non-smoker	43 (71.7%)	44 (73.3%)	0.838
	Smoker	17 (28.3%)	16 (26.7%	
Contraception	None	51 (85.0%)	49 (81.7%)	0.829
	IUD	7 (11.7%)	7 (11.7%)	
	OCP	2 (3.3%)	4 (6.7%)	
Arthralgia		41 (68.3%)	35 (58.3%)	0.256
Oral ulcers		57 (95.0%)	18 (30.0%)	< 0.001*
HTN		23 (38.3%)	23 (38.3%)	0.897
Pathergy test		45 (75.0%)	1 (1.7%)	< 0.001*

^*^Significant

On comparing laboratory investigations between the two studied groups, there was a significant increase in serum levels of BUN, anti-tubulin-alpha-1c, and ESR (Table 2) and significant decrease in eGFR and C3 in BS patients compared to healthy controls. Urine analysis in BS patients detected significant increase in pus cells, p/c, casts, and albuminuria compared to healthy controls as shown in Table 2.

**Table 2:** Laboratory data of both studied groups

	Cases	Controls	*p* value
	Mean ± SD	Mean ± SD	
White blood cells	7.1** ± **3.6	7.1** ± **3.6	1.000
Hemoglobin	10.1** ± **1.8	12.9** ± **1.1	1.000
Platelets	217.7** ± **108.3	231.5** ± **70.5	0.413
Blood urea nitrogen (BUN)	26.1** ± **6.5	16.4** ± **5.0	< 0.001*
Creatinine	0.9** ± **0.3	1.0** ± **0.8	0.460
C3	89.6** ± **38.1	128.9** ± **26.8	0.000*
C4	26.4** ± **12.0	28.4** ± **10.6	0.331
Erythrocyte sedimentation rate	86.8** ± **42.6	28.9** ± **24.4	0.000*
C-reactive protein	13.7** ± **18.0	11.4** ± **9.8	0.380
Pus cells	6.1** ± **5.9	4.0** ± **2.7	0.013*
Albumin	0.3** ± **0.4	0.0** ± **0.0	0.000*
Casts	0.1** ± **0.3	0.0** ± **0.0	0.022*
Protein/creatinine ratio	5.5** ± **10.5	2.0** ± **2.7	0.015*
Anti-endothelial cell antibodies	44.7** ± **12.6	41.0** ± **8.8	0.064
Anti-tubulin-alpha-1c antibody	76.9** ± **21.7	38.0** ± **9.9	0.000*

^*^Significant

Regarding correlations of AECA with different clinical presentations among cases, there was no significant correlation among any except with venous thrombosis (*p* value 0.024), while the correlations of anti-tubulin-alpha-1c with different disease presentations among cases show significant direct correlation with posterior uveitis, panuveitis, and venous thrombosis with *p* values of 0.023, 0.034, and 0.009, respectively.

On analyzing correlations of AECA with different disease parameters among cases, we found no significant correlation among any except with CRP with *p* value 0.032 as shown in Table 3. Table 4 shows the correlations of anti-tubulin-alpha-1c with different disease parameters among cases in which there is significant direct correlation with BVAS, C4, and p/c.

**Table 3:** Correlations of anti-endothelial cell antibodies (AECA) with disease duration, Behçet syndrome (BS) activity, Birmingham Vasculitis Activity Score (BVAS), and laboratory data among cases

		AECA
Disease duration	Pearson correlation	0.035
	*p* value	0.789
BVAS score	Pearson correlation	0.141
	*p* value	0.282
Behcet activity score	Pearson correlation	0.091
	*p* value	0.490
White blood cells	Pearson correlation	− 0.159
	*p* value	0.224
Hemoglobin	Pearson correlation	− 0.069
	*p* value	0.600
Platelets	Pearson correlation	0.164
	*p* value	0.211
Blood urea nitrogen	Pearson correlation	0.207
	*p* value	0.113
Creatinine	Pearson correlation	− 0.132
	*p* value	0.316
ANA	Pearson correlation	0.161
	*p* value	0.220
Anti-DNA	Pearson correlation	0.041
	*p* value	0.757
ANCA	Pearson correlation	0.088
	*p* value	0.506
C3	Pearson correlation	0.020
	*p* value	0.882
C4	Pearson correlation	− 0.134
	*p* value	0.306
Erythrocyte sedimentation rate	Pearson correlation	0.054
	*p* value	0.683
C-reactive protein	Pearson correlation	277(*)
	*p* value	0.032
Pus cells	Pearson correlation	− 0.024
	*p* value	0.856
Albumin	Pearson correlation	0.170
	*p* value	0.194
Casts	Pearson correlation	− 0.032
	*p* value	0.808
Protein/creatinine ratio	Pearson correlation	− 0.028
	*p* value	0.829

^*^Significant correlation

**Table 4:** Correlations of anti-tubulin-alpha-1c with disease duration, Behçet syndrome (BS) activity, Birmingham Vasculitis Activity Score (BVAS), and laboratory data among cases

		Anti-tubulin-alpha-1c
Disease duration	Pearson correlation	0.012
	*p* value	0.930
BVAS score	Pearson correlation	0.198
	*p* value	0.129
Behcet activity score	Pearson correlation	.385(**)
	*p* value	0.002
White blood cells	Pearson correlation	0.245
	*p* value	0.059
Hemoglobin	Pearson correlation	0.017
	*p* value	0.897
Platelets	Pearson correlation	0.140
	*p* value	0.286
Blood urea nitrogen	Pearson correlation	0.200
	*p* value	0.126
Creatinine	Pearson correlation	− 0.171
	*p* value	0.193
ANA	Pearson correlation	0.138
	*p* value	0.294
Anti-DNA	Pearson correlation	− 0.114
	*p* value	0.388
ANCA	Pearson correlation	− 0.047
	*p* value	0.721
C3	Pearson correlation	− 0.082
	*p* value	0.535
C4	Pearson correlation	− .266(*)
	*p* value	0.040
Erythrocyte sedimentation rate	Pearson correlation	0.235
	*p* value	0.070
C-reactive protein	Pearson correlation	0.103
	*p* value	0.434
Pus cells	Pearson correlation	− 0.191
	*p* value	0.144
Albumin	Pearson correlation	0.180
	*p* value	0.169
Casts	Pearson correlation	0.046
	*p* value	0.726
Protein/creatinine ratio	Pearson correlation	.275(*)
	*p* value	0.033

^*^Significant correlation

^**^Highly significant correlation

**Fig. 2:**
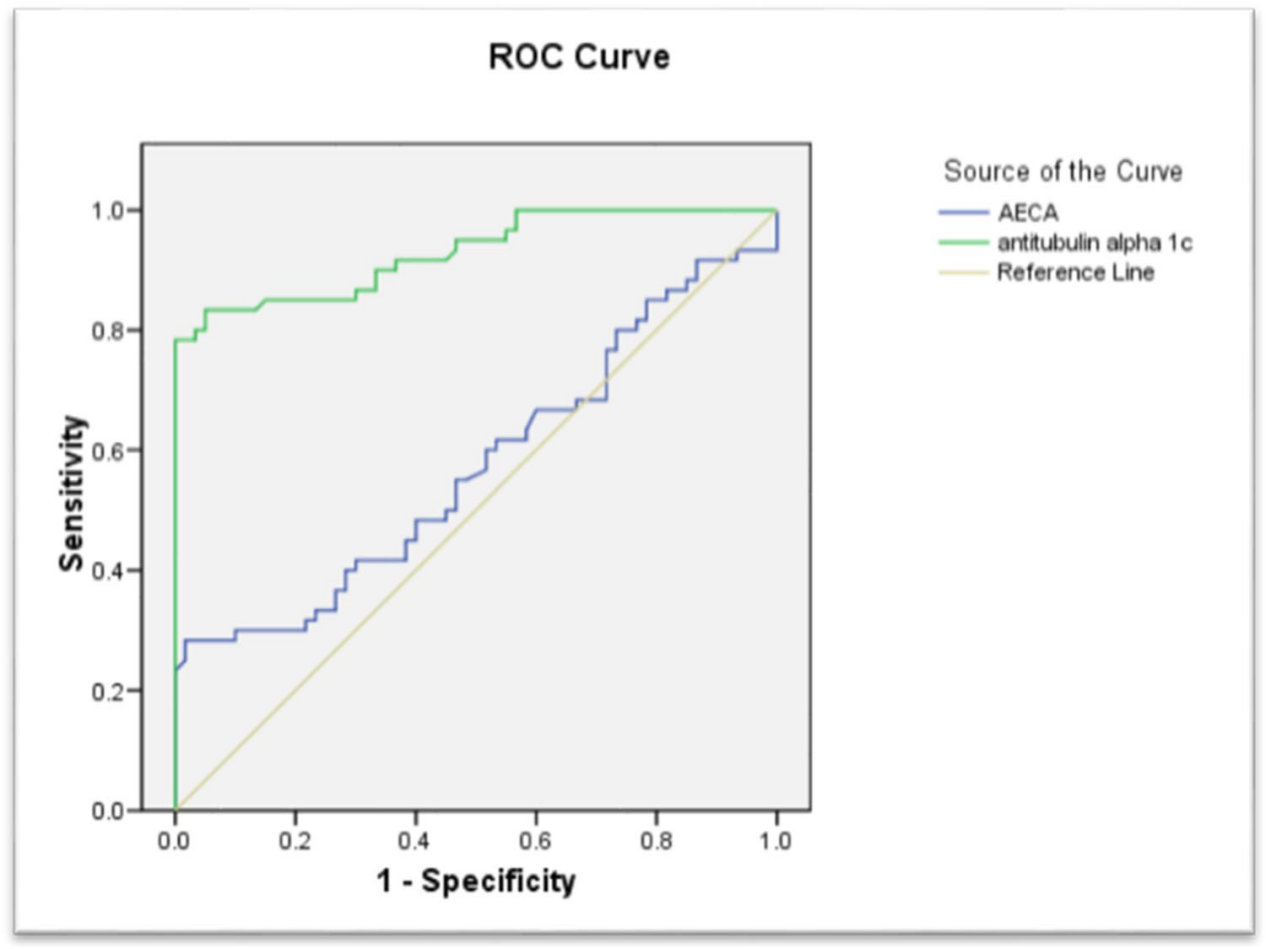
ROC curve for diagnostic performance of anti-endothelial cell antibodies (AECA) and anti-tubulin-alpha-1c

On analyzing the diagnostic performance of both AECA and anti-tubulin-alpha-1c, ROC analysis detected that the cutoff value of the AECA for diagnosis was 27.250, with sensitivity and specificity of 93.3% and 96.7%, respectively, while the cutoff value of anti-tubulin-alpha-1c for diagnosis was 22.300, with sensitivity and specificity of 100% and 96.7%, respectively, as shown in Fig. 2.

## Discussion

Behçet’s syndrome (BS), as an autoimmune vasculitis with multi-organ involvement, has no specific laboratory investigations for diagnosis. There may be increase in inflammatory markers as C-reactive protein and erythrocyte sedimentation rate during activity [32]. Detection of autoantibodies is the cornerstone of various autoimmune diseases’ diagnosis. However, BS lacks any specific autoantibodies. Hence, it is essential to detect autoantibodies as diagnostic biomarkers. Several potential autoantigens were detected in circulating immune complexes of BS patients which were involved in pathophysiological abnormalities of BS including immune response, cell structure integrity, and coagulation cascade [33].

Previous studies detected that anti-endothelial cell autoantibody (AECA) has contributed to vascular damage in BS [17, 34, 35]. However, AECA positivity percentages detected were low in different BS studies, which were 13.1%, 26%, and 47.5% in Turkish, Spanish, and Chinese patients with BS, respectively. In addition, AECA was also detected in healthy participants, so lacking its sensitivity and specificity in BS [15, 35]. Elevation of anti-tubulin autoantibodies is attributed to autoimmune reactions to the patient’s own tubulin. However, specific autoantibodies against α-tubulin, one of the tubulin molecular members, were only observed in patients with chronic allograft rejection after lung transplantation in some studies [36, 37], and its prevalence and clinical significance have not been determined yet in autoimmune diseases. To our knowledge, few studies had assessed this. Hence, we aimed to evaluate the significance of the autoantibody against tubulin-α-1c in BS.

Our study was conducted on sixty patients diagnosed to have BS and sixty healthy individuals who served as a control group. Both groups were comparable for age and gender. From the sixty BS patients, 55% were with high disease activity score and 45% with low disease activity score. On comparing laboratory investigations, there was a significant increase in anti-tubulin-alpha-1c and ESR with significant decrease of C3 in patients compared to control. Urine analysis in cases detected significant increase in pus cells, p/c, casts, and albuminuria compared to healthy controls as expected. This is consistent with Cheng et al. (2018) who confirmed that tubulin-α-1c was significantly increased in 44 patients with BS than those with other autoimmune diseases and healthy controls. However, our study included only patients with BS and healthy controls [33].

Natural autoantibodies to tubulin, the basic unit of microtubules, are present in healthy humans [38] in low titers [39]. However, elevated levels of serum anti-tubulin autoantibodies have been reported to be associated with organ-specific autoimmune diseases as Graves’ disease [40]. Tubulins are involved in the maintenance of cytoskeleton including both the cellular structure and intracellular movement [40]. It was postulated that tubulin-α-1c might also stimulate the expression of vascular endothelial growth factor and damage the endothelial cells resulting in vasculitis and thrombosis [41].

In the present study, there was non-statistically significant increase in serum AECA in BS patients compared to healthy controls. This is in contrast to Zheng et al. (2005) who studied AECA in BS and other autoimmune diseases and detected that AECA was found more frequently in patients with BS, especially with increased disease activity, and concluded that it can be used in monitoring disease activity and effect of therapy. This contrast can be explained by the fact that Zeng and collages examined the level of AECA at the activity period of disease compared to the controlled period. However, 55% of our patients had high disease activity index, and 45% were with low disease activity index. Unfortunately, AECA could also be found in other systemic vasculitis and SLE [35].

Endothelial cell dysfunction [34] and vasculitis [42] are major pathological findings in BS. Although the etio-pathogenesis of the BS is still unknown, anti-endothelial cell antibodies (AECAs) may be involved in the pathogenesis of vascular injury which is the main cause of autoimmune diseases [43]. But in the current study, non-significant increase was detected in BS patients, and this may be related to AECA positivity in healthy controls, making its sensitivity and specificity for diagnosis of BS very challenging [15, 35]

On analyzing correlations of AECA with different disease presentations among cases, there was non-significant correlation among any except with venous thrombosis (*p* value 0.024), while regarding correlations of anti-tubulin-alpha-1c with different disease presentations among cases, there is significant direct correlation with posterior uveitis, panuveitis, and venous thrombosis with *p* values of 0.023, 0.034, and 0.009, respectively. In line with ours, Chen and colleagues detected that anti-tubulin-α-1c was associated with complications of deep venous thrombosis and erythema nodosum in BD [33]. However in ours, there is no association with any dermatological but with ophthalmological and vascular complications.

Regarding correlations of AECA with different parameters among cases, there was non-significant correlation except of CRP with *p* value 0.032, while regarding correlations of anti-tubulin-alpha-1c among cases, there is significant direct correlation with BVAS, C4, and p/c. In harmony with ours, Cheng et al. (2018) showed that anti-tubulin-α-1c autoantibodies were significantly positively correlated with BS disease activity but also with inflammatory markers ESR and CRP.

To the best of our knowledge, ROC curve for both AECA and anti-tubulin-alpha-1c in BS was done for the first time in the current study. Regarding the diagnostic performance of both AECA and anti-tubulin-alpha-1c, the cutoff value of the AECA for diagnosis was 27.250, with sensitivity and specificity of 93.3% and 96.7%, respectively, while the cutoff value of the anti-tubulin-alpha-1c for diagnosis was 22.300, with sensitivity and specificity of 100% and 96.7%, respectively.

Previous research reported that immune complex deposition, which is formed by vascular autoantigens such as histones, ribosomes, fibronectin, and their autoantibodies, was able to induce exacerbated local inflammation within the vascular endothelium. Anti-tubulin-α-1c might bind to its antigen on vascular cells and induce inflammatory response and exacerbate tissue damage [44, 45]. However, the exact role of the pathogenesis of anti-tubulin-α-1c in BS is still to be determined by further research, to identify the pathogenic roles of anti-tubulin-α-1c in BS.

## Conclusion

Identification of anti-tubulin-α-1c antibodies as a novel biomarker for BS diagnosis may be promising for better targeting therapy. Elevation of serum tubulin-α-1c autoantibody was detected in BS with sensitivity and specificity of 100% and 96.7%, respectively. The anti-tubulin-α-1c autoantibody was positively correlated with venous thrombosis, uveitis, and disease activity in BS, which indicated liability of its involvement in the vascular pathogenesis of BS. Larger cohort studies for exploration of the exact roles of tubulin-α-1c and its autoantibodies are required.

## Supplementary Information

Below is the link to the electronic supplementary material.

Supplementary file1 (DOC 50 kb)

## Data Availability

The data underlying this article will be shared on reasonable request to the corresponding author.
