# Supplementary figures and images for: Anti-tubulin-alpha-1c antibody as a marker of value in Behçet syndrome
Source: Clin Rheumatol. 2022 Feb 7;41(6):1759–67. doi: 10.1007/s10067-021-06025-7 (PMC9119891; doi:10.1007/s10067-021-06025-7)

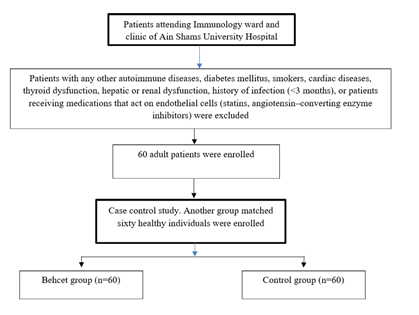

Supplement: Supplementary file 1 — Supplementary file1 (DOC 50 kb) [file 10067_2021_6025_MOESM1_ESM.doc]
